# Re-evaluation of the prospective risk analysis for artificial-intelligence driven cone beam computed tomography-based online adaptive radiotherapy after one year of clinical experience

**DOI:** 10.1016/j.zemedi.2024.05.001

**Published:** 2024-06-08

**Authors:** Sonja Wegener, Paul Käthner, Stefan Weick, Robert Schindhelm, Kathrin Breuer, Silke Stark, Heike Hutzel, Paul Lutyj, Marcus Zimmermann, Jörg Tamihardja, Andrea Wittig, Florian Exner, Gary Razinskas

**Affiliations:** University Hospital Würzburg, Department of Radiotherapy and Radiation Oncology, Josef-Schneider-Str. 11, 97080 Würzburg, Germany

**Keywords:** risk analysis, online adaptive, FMEA, Ethos

## Abstract

Cone-beam computed tomography (CBCT)-based online adaptation is increasingly being introduced into many clinics. Upon implementation of a new treatment technique, a prospective risk analysis is required and enhances workflow safety. We conducted a risk analysis using Failure Mode and Effects Analysis (FMEA) upon the introduction of an online adaptive treatment programme (Wegener et al., Z Med Phys. 2022).

A prospective risk analysis, lacking in-depth clinical experience with a treatment modality or treatment machine, relies on imagination and estimates of the occurrence of different failure modes. Therefore, we systematically documented all irregularities during the first year of online adaptation, namely all cases in which quality assurance detected undesired states potentially leading to negative consequences. Additionally, the quality of automatic contouring was evaluated. Based on those quantitative data, the risk analysis was updated by an interprofessional team. Furthermore, a hypothetical radiation therapist-only workflow during adaptive sessions was included in the prospective analysis, as opposed to the involvement of an interprofessional team performing each adaptive treatment.

A total of 126 irregularities were recorded during the first year. During that time period, many of the previously anticipated failure modes (almost) occurred, indicating that the initial prospective risk analysis captured relevant failure modes. However, some scenarios were not anticipated, emphasizing the limits of a prospective risk analysis. This underscores the need for regular updates to the risk analysis. The most critical failure modes are presented together with possible mitigation strategies. It was further noted that almost half of the reported irregularities applied to the non-adaptive treatments on this treatment machine, primarily due to a manual plan import step implemented in the institution’s workflow.

## Introduction

Online-adaptive radiotherapy enables the adjustment of treatment plans to accommodate interfractional anatomical changes, offering the potential to reduce margins around target structures beside other expected benefits. Besides online-adaptation on linear accelerators with magnetic resonance imaging (MRI) capabilities, the Varian Ethos system facilitates online-adaptive radiotherapy based on cone beam computed tomography (CBCT) images. [1] The Ethos system is utilized for a variety of treatment sites, predominantly in the pelvic area, including rectum [2], bladder [2], [3] and prostate [4], next to other treatment sites.

The introduction of online-adaptive radiotherapy necessitates the implementation of new workflows, distinct from the offline adaptive workflow and non-adaptive, image-guided radiotherapy (IGRT) workflows at the treatment machine that are well established at most institutions. [5], [6], [7] New workflows introduce new failure modes, as identified in prospective risk analyses for both the MRI- [5], [8] and the CBCT-based [6], [9], [10] technologies.

Recommendations emphasize the necessity for a prospective risk analysis by an interdisciplinary team upon the implementation of a new treatment technique, as well as a periodic revision of the results. [11] A 2021 survey in Germany found that around half of the participating centres had updated their risk analysis at least once. [12] A report on the reassessment of the radiotherapy workflow after 10 years emphasizes the value of regular updates. [13] Therefore, further insights can also be anticipated from a repeat analysis of the new technology-driven workflow of online-adaptive radiotherapy. Results of such an analysis including the first year experience of a single center are published for the MRI-guided workflow. [14] First year experiences of the CBCT-based online adaptive workflow on Ethos have been provided with a focus on questions arising during the implementation of an adaptive programme. [15] Only very few risk analyses of the CBCT-based online-adaptive process on Ethos have been published and, to the best of our knowledge, there has been no re-evaluation. To include relevant failure modes, individual users must rely on their own experience or literature data. While many failure modes as well as their frequency of occurrence are published for traditional non-adaptive radiotherapy workflows [16], [17], such information is also vital for online adaptive radiotherapy.

We updated our previous analysis of the Ethos adaptive workflow [6] after one year of clinical experience. The re-evaluation aims to achieve three goals. First, from a clinical perspective, it should improve the workflow, maximizing safety for treatments. Second, from a risk management perspective, the study emphasizes the value of a prospective risk analysis conducted before the first clinical experience with a new treatment machine by illustrating how the initial study and the re-evaluation differ. Third, from the perspective of an adaptive radiotherapy user, the study offers insights into how risks and staff roles are interconnected, providing a rationale for the staff required at adaptive treatments.

## Material and methods

### Preparations

We conducted a prospective risk analysis during the introduction of an Ethos treatment machine (hardware version Halcyon 3.1 and software version Ethos Treatment Management 2.1, Varian Medical Systems, Palo Alto, CA, USA) in spring 2022. [6] A total of 122 failure modes were identified using a Failure Mode and Effects Analysis (FMEA) approach. Working with a new treatment system, all input for this prospective risk analysis relied on points addressed in training or experienced during test runs. This limitation was previously acknowledged in our study and prompted a re-evaluation after one year of clinical experience.

**Figure 1:**
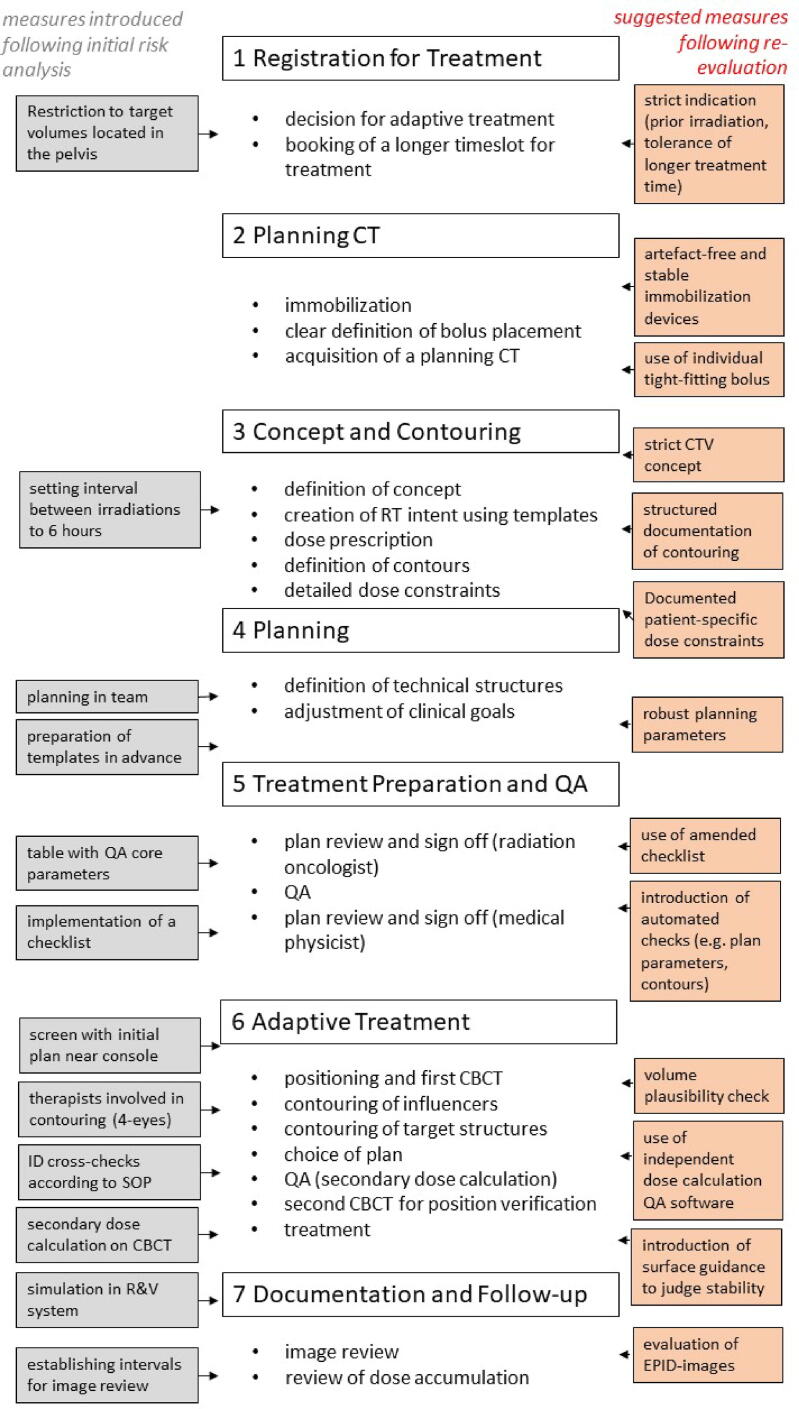
Schematic depicting the online-adaptive workflow on Ethos that is evaluated. Measures implemented following the initial risk analysis are presented on the left. Recommended measures derived from re-evaluation are presented on the right. Abbreviations: EPID – electronic portal imaging device, R&V system – Record & Verify system, SOP – standard operating procedure.

The online adaptive treatment programme commenced in spring 2022 at our institution. Following our initial analysis, the workflow depicted in Fig. 1 was implemented and maintained with minimal variability during the first year of treatments. As a consequence of the initial risk analysis, the first period of the adaptive programme was restricted to the pelvic region as part of a risk mitigation strategy. Throughout the first year, 400 fractions were delivered following the adaptive workflow. Additionally, quality assurance and phantom irradiations were performed to enhance understanding of the Ethos system, especially the behaviour of software algorithms in distinct clinical situations.

To systematically capture further possible failure modes and assign empirical values for their occurrence, all users were encouraged to report all irregularities into a browser-based entry mask, accessible from all computers within the hospital, including a computer next to the treatment machine. We emphasized that entries should also include “good catches” of errors noted before the treatment, within or beyond the scope of the regular quality assurance steps. Additionally, after each fraction, the effort for manual editing of influencers and target structures, as well as comments regarding the contouring and dose distribution, were recorded in a spreadsheet.

### Evaluated adaptive workflow

The institutiońs current clinical protocol for online adaptive radiotherapy on Ethos closely adheres to the standard Ethos workflow [1] and is described also in [6]. During the adaptation, a team of radiation oncologist, medical physicist and radiation therapists is present at the machine. The radiation oncologist reviews the CBCT images, organs and target structures proposed by the machine and chooses the treatment plan. The medical physicists verifies the plan dosimetrically by secondary dose calculation using Mobius software before the plan is loaded for treatment. Due to the time elapsed since the first CBCT, a second CBCT is performed to monitor for intrafractional changes and to perform translational corrections when necessary. In this workflow, radiation therapists are responsible for patient positioning, observation of patient movements and the operation of the treatment machine including image acquisition and treatment delivery. All team members will usually observe the contouring and the plan review step.

Additionally, the feasibility of a hypothetical radiation therapists-only workflow was examined. In the following, a therapist-only workflow consists of a first treatment with a radiation oncologist, two radiation therapists and a physicist at the machine briefing the radiation therapists. All subsequent fractions are independently conducted by the two radiation therapists, with the option to call either a physicist or a radiation oncologist for assistance if needed. The center has no practical experience with the above-mentioned therapist-only workflow. The proposed workflow is solely evaluated by means of a prospective risk analysis.

### Risk analysis for the adaptive workflow

The initial analysis was integrated into the risk management software myQA PROactive (version 1.14, iba Dosimetry, Schwarzenbruck, Germany). A risk management group was compiled, comprising members of all professions involved in the online adaptive programme. The group included the same team as during the initial evaluation: three radiation oncologists, four physicists and two radiation therapists, augmented by a physicist who, while not involved in the adaptive programme, moderated the sessions. The group convened seven times for approximately one hour.

For a re-evaluation of the workflow using FMEA, all steps in the workflow were reviewed against the current clinical protocol. Failure modes from the initial analysis were either retained or deemed irrelevant, and additional failure modes were incorporated based on recorded irregularities, personal experience, and literature studies.

Group members and other experienced Ethos users within the institution were invited to score the status quo workflow, which involved a team of a radiation oncologist, physicist, and two radiation therapists at the machine for each adaptive fraction. Scores for the therapist-only workflow could be entered in distinct rows in the same main scoring document. Scores for severity (S), occurrence (O), and detectability (D) were entered individually on a scale from 1 to 10 (details in Appendix A). We adjusted the scale between the initial evaluation and the re-evaluation for practical reasons. The currently used scale is adapted from the scale presented by S. Weigt and S. Menkel in a risk management working group meeting in Dresden in 2022. Participants were encouraged to score only those items they felt confident in assessing. When unable to provide a score for a particular situation, they were instructed to leave a blank scoring field, such that only confident scores were included in the further analysis.

Eight scoring sheets were returned, including the assessment from all three professions. Mean values of severity (S), occurrence (O), and detectability (D) were calculated. The risk priority number (RPN) was derived from the mean values as follows:

(1) RPN=S∗O∗D.

All failure modes were ranked according to their RPN. For the 20 highest failure modes, additional preventions (measures that prevent/reduce the possibility of occurrence) and barriers (measures that improve the detectability) were discussed in the group. In a second evaluation round, participants indicated how the introduction of each additional measure would impact their scoring. For the second evaluation round, two of the previous scorers were not available. Six evaluation sheets were returned. The number of 20 failure modes was picked as a manageable number, aiming to address the most urgent points together and consider further points in smaller groups or in the next iteration of the evaluation.

Summed up, the seven hours of face-to-face meetings and approximately two hours per person spent scoring total to around 100 hours for the team. In addition, thorough preparation of each session was performed by the project administration in the background, which we estimate to have taken just as long.

## Results

### Recorded Irregularities for the adaptive workflow

Within the first year of operating the Ethos, 126 irregularities were reported, with 66 related to adaptive treatments. The irregularities can be categorized into six groups: issues with contouring, human or workflow errors, treatment planning challenges, changes in the patient anatomy, machine limitations, and machine faults. A complete list of the irregularities is provided in Table 1.

**Table 1:** Recorded irregularities during the first year of adaptive treatments using the Ethos. The majority of the irregularities listed constitute “good catches”, identified through routine quality assurance.

**Contouring**	**Human or workflow errors**	**Treatment planning**
• suggested target volume delineation proved insufficient: rigid propagation yields better results (24×) • lymph nodes misidentified as bowel • bladder as an influencer not adequately covered in CBCT (3×) • replanning with additional influencers necessary to yield acceptable plan	• patient empties bladder against instructions due to extended waiting time • (second) CBCT directly before treatment was omitted (3×) • name of target volume absent from Mobius dictionary • unintended revision renders the approved plan unusable • derived structures turned into static contours • prepared QA folder deleted	• target volume extends beyond the capacity of a single isocenter only during adaptive session • excessively high dose within or adjacent to the target volume (4×) • adapted plan could not be generated
**Changes in patient anatomy**	**Machine limitations**	**Machine faults**
• extreme changes in bladder or rectum filling (9×) • alteration of PTV position (4×) • presence of excessive rectal gas (2×) • immobilization lacks stability	• CBCT artefacts complicate contouring • immobilization device induces image artefacts • bladder as an influencer challenging to identify (2×) • fusion with MR images not visible unless contours are edited	• software unavailable for an extended period • machine unexpectedly shuts down

While some of the irregularities were to be expected from the initial prospective risk analysis, others were unexpected and warrant further investigation:

It occurred that a target volume just fitted into the field size of a one-isocenter plan during initial plan generation. However, during the adaptive session the patient shifted slightly, or the target volume extended over a longer cranio-caudal distance due to anatomical changes. In that case, the adapted treatment plan dose did not sufficiently cover the target volume and thus became unusable. The machine is unable to add a second isocenter for a larger treatment volume if this was not specified in the initial plan. This factor should be considered for target volumes approaching the length of the treatment field.

There were (rare) instances when the machine failed to calculate an adapted plan. In such situations, reverting to the initial plan is possible, but this may compromise the treatment if margins are reduced for adaptive treatments. Attention should also be drawn to the fact that a few unintended clicks can convert derived structures into static ones. This means that composite structures are not recalculated from contoured targets or influencers each time but remain static as in the initial treatment plan. This may yield undesired dose distributions in the adapted plan but can be easily identified during plan review if one is aware of this possible error.

CBCT directly before the treatment account for positional changes during the adaptation. The applied translation vectors had an average length of 2.0 mm (± 1.4 mm) evaluated over 270 adaptive sessions. There were some instances, when those CBCTs were omitted. Moreover, we observed that one of our immobilization devices with a very steep edge caused artefacts in the CBCT. While this is inconsequential for non-adaptive IGRT treatments, it poses challenges with contouring in the respective slices during adaptive treatments.

### Failure Modes for the adaptive workflow

**Figure 2:**
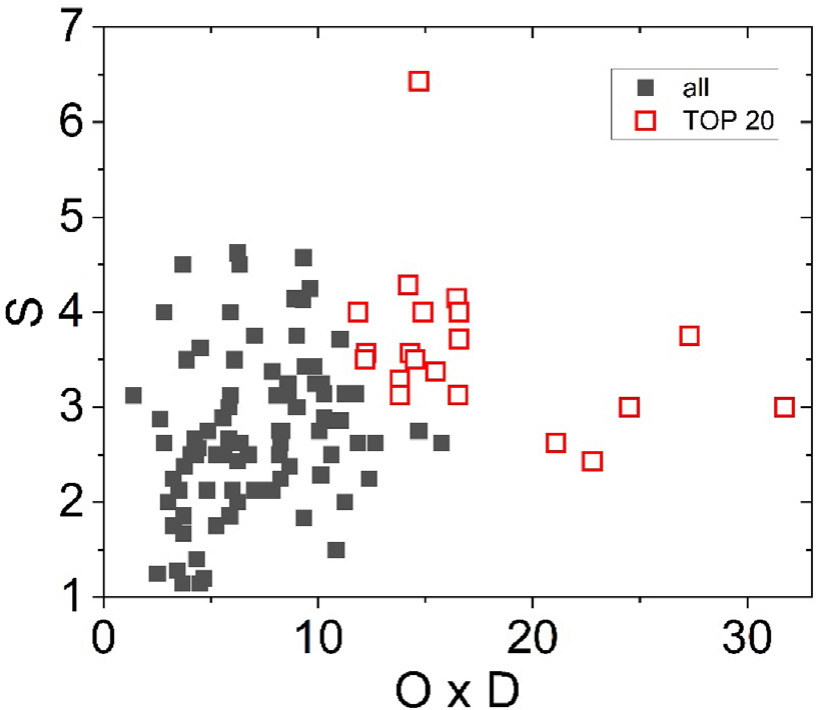
Risk matrix including all 111 failure modes. The TOP 20 with the highest RPN are displayed as red unfilled squares and were investigated more thoroughly.

A list of 111 failure modes was compiled, representing a smaller number than what was identified in the initial evaluation. Some failure modes, having similar causes and consequences, were merged into fewer, broader failure mode categories. These concern, for example, appointment booking or requirements of the planning CT. Newly recorded irregularities were included in the list. A matrix depicting occurrence multiplied by detectability versus severity for all failure modes is displayed in Fig. 2.

The 20 highest-ranked failure modes are tabulated (Table 2). Failure modes either occurred, almost occurred, or are merely hypothetical. It can be noted that failure modes identified as “new in re-evaluation” (almost) occurred and were, therefore, added to the analysis. The list of highest ranked failure modes has changed compared to the initial evaluation due to three main reasons. Firstly, some of the risks have been mitigated and new workflow steps have been included. Although these steps improved the initial situation, they also introduced additional risks (TOP 2 and TOP 6). Secondly, certain high-ranked risks from the previous evaluation are no longer a major concern. For instance, standardization of isodose display and user familiarity with it over the first year reduced its priority. While it was previously ranked 6, it now holds a lower RPN. Density changes and associated issues with the synthetic CT are not as prominently ranked due to our institution’s workflow being limited to the pelvic region. Finally, some of the items have been scored differently, as the number of observed problems surpassed the anticipated occurrence within the first year. As a notable example, issues with the planning process and templates have moved up the ranks.

**Table 2:** The 20 highest-ranked failure modes. Where applicable, the rank in the initial evaluation, the RZN in the therapist-only workflow, countermeasures are indicated. Identified countermeasures, comprising added preventions and barriers, are listed along with an indication of their impact.

**TOP**	**Step**	**Failure Mode**	**RPN**	**initial rank**	**RPN therapist-only workflow**	**Suggested additional preventions/** **barriers**	**RPN after imple-mentation**
1	Irradiation, Adaption	patient details not known to advanced adaptor, resulting in adaption not utilizing the best possible contours	**102.4**	7	82.9	additional step in workflow: contour review in team	64.7
						structured documentation of contouring process and related information	61.1
2	Irradiation, Documentation	treated fraction not manually simulated to record the delivered dose in the record and verify system or manually simulated fraction not irradiated, leading to incorrect documentation with the risk of delivering an incorrect number of fractions and incorrect total delivered dose	**95.2**	-		software update required	19.7
3	Contouring, Prior Irradiation	summation of current plan with dose from prior irradiation not possible, leading to an incorrect consideration of the prior dose	**94.5**	2		transfer of initial plan into Eclipse for dose summation	68.9
						strict indication of adaptive treatments	64.3
4	Preparations, Physics QA	QA tolerances unable to detect errors, leading to the irradiation of a technically incorrect plan with an incorrect dose	**73.5**	12		adopt different secondary dose calculation software	48.0
						systematic analysis of sensitivity on phantom study	41.1
5	Contouring,RT intent	wrong or outdated template used for planning, leading to an incorrect treatment or an incorrect dose distribution during adaptation	**68.1**	^			
6	Irradiation, Documentation	skipped fraction manually simulated at the time of the next treatment, leading to incorrect documentation with the risk of delivering an incorrect number of fractions	**66.3**	-		software update required	35.3
7	Contouring, Planning Directives	no template available, spontaneous input of values, leading to an incorrect dose distribution during adaptation	**61.6**	19		creation of templates prior to introducing new entities	30.8
8	Contouring, Preliminary Concept	concept not correctly entered in ARIA, resulting in it being wrongly copied to the Ethos software, leading to incorrect timing of plans or the omission of boost treatment	**60.9**	15		documentation of concept, Care Path for boost treatment to be created upon start of treatment	29.4
9	Contouring,RT intent	template not corresponding to irradiation type specified in the preliminary concept, leading to an incorrect dose distribution during adaptation	**59.5**	^			
10	Plan generation, Dose Preview	manual adjustment of DVH curves, with changes only applied in initial plan, leading to an incorrect dose distribution during adaptation	**55.4**	^			
11	Plan Generation, authorization	saving a dose preview, resulting in lower quality plans	**55.3**	^			
12	Plan Generation, Dose Preview	incorrect prioritization of goals, leading to an incorrect dose during adaptation	**52.2**	^			
13	Plan Generation, Dose Preview	prioritization not customized to individual patient when necessary, leading to an incorrect dose during adaptation	**51.6**	^		document relevant patient-specific dose constraints alongside preliminary concept	25.0
14	Planning CT, Acquisition	highly variable or non-persistent air bubbles, leading to issues with synthetic CT and dose calculation	**51.0**	^		dose calculation on CBCT, hardware and software update, dose recalculation on CBCT	7.9
15	Irradiation, Adaption	incorrect edits on target structures by advanced adaptor, leading to an incorrect dose distribution	**50.8**	16	47.8	plausibility check of target structures, e.g. comparison with earlier fractions	44.4
						introduction of a four-eyes principle, also involving radiation therapists	48.6
16	Irradiation, Imaging	substantial anatomical changes, including density variations, affecting the synthetic CT, leading to dose calculation issues	**47.4**	4		dose calculation on CBCT, hardware and software update, dose recalculation on CBCT	18.9
17	Contouring, RT Intent	treatment region already in use, necessitating the use of an alternative region, leading to documentation issues and the potential for irradiating an incorrect plan	**45.3**	^			
18	Contouring, Planning Directives	individual goals omitted, leading to a comprised plan quality during adaption	**43.7**	^		introduce automated plan check using software	21.3
19	Irradiation, Adaption	software-generated target volumes incorrect	**43.2**	^	46.4	implementation of a robust CTV concept	14.3
20	Irradiation, Adaption	excessively high dose within or outside the target volume in the newly optimized plan	**42.7**	^			

- : new in re-evaluation,^moved up from a rank below 20 in the initial analysis.

Mitigation strategies for the TOP 20 failure modes were discussed. All identified preventions and barriers are included in Table 2. Suggested workflow modifications are illustrated in Fig. 1. Beyond the TOP 20, we found that reliable positioning in immobilization devices minimizes intrafractional changes. Therefore, the use of positioning devices should also be reassessed for adaptive radiotherapy applications. Surface guidance will quantitatively monitor patient movement and we plan to combine it with adaptive radiotherapy as a barrier to detect the need for repositioning.

### Radiation therapist-only adaptive workflow

For 20 failure modes, changes in the workflow occur when transitioning from the current status, which involves an interdisciplinary team present at each adaptive fraction, to a hypothetical therapist-only workflow. These aspects were individually scored for the alternative workflow. Mean differences in RPN of each failure mode between the two workflows are 1.0 with one standard deviation of 5.1. The median is 0.3. Positive numbers indicate that the status quo workflow has a higher RPN than the therapist-only workflow. The distribution is almost symmetric around the origin, suggesting that the overall risk does not change when adopting a therapist-only workflow. The most substantial reduction of almost 20 points was noted for the failure mode identified as TOP 1 (Table 2).

### IGRT treatment on Ethos

Although not specific to online adaptive treatments, several Ethos-specific irregularities were recorded for non-adaptive IGRT as well. A complete list of the 56 irregularities observed in the IGRT-workflow with the Ethos system is available in Table 3.

**Table 3:** Irregularities reported for non-adaptive treatments on Ethos. Most of the listed irregularities are “good catches”, which were detected during standard quality assurance.

**Wrong plan**	**Documentation**	**Machine limitations**
• reoptimized plan chosen for irradiation instead of imported plan • more than one plan available for irradiation on Halcyon while Ethos not operational • QA performed for wrong plan only • wrong plan entered into QA process • new plan added sequentially instead of replacing the existing plan in the current series	• manual simulation in ARIA not performed (27×) • manual simulation of skipped fraction performed nonetheless • treatment series closed with no change in concept • manual simulation listed under wrong treatment machine on daily lists • unused RT intents remain unremoved • imported into incorrectly labelled region (4×)	• isocenter too far from reference point in cranio-caudal direction; couch does not move (2×) • plan cannot be loaded on treatment machine due to presence of specific structures (software bug)
**Import**	**Others**	
• non-adaptive plan labelled as adaptive (2×) • interval assignment (e.g. daily) incorrect (2×) • inaccurate bolus information • delta couch shifts not correct in Ethos (2×) • incorrect contour used for PTV in Ethos • exclusion of relevant contours in Ethos • plan sign-off hindered due to incorrect assignment of target contours • parallel boost entered incorrectly as a sequential series	• incorrect CarePath entries after patient transitions to a different treatment machine • localization point incorrectly selected in Eclipse • field order suboptimal and unmodifiable once plan is imported into Ethos software (2×) • CT performed from incorrect reference point • approved plans become invalid following modifications to technical structures	

Very briefly, the workflow for non-adaptive patients includes planning in Eclipse, followed by a treatment plan import into the Ethos software. Following the import, the Ethos software always reoptimizes an imported plan, resulting in multiple plans being available in the software, thus increasing the risk of selecting the incorrect plan for treatment. Among the listed irregularities, five instances were noted where the wrong plan could have been mistakenly chosen. In addition, the import process is prone to entering wrong details. Foremost, there needs to be clarity on how a new plan is integrated into the system. The Ethos system follows a concept of defining “phases”, upon creation of which the user needs to correctly determine whether a new plan is added to the treatment course or intended to replace the currently used plan.

The aforementioned import step is only necessary when planning in Eclipse and transferring plans to the Ethos software afterwards. This institution has decided to include the extra step due to planning experience and more freedom in choosing planning parameters in Eclipse as well as the desire to have better interoperability with and workflows mostly comparable to a a Halcyon linac in the same institution.

The treatment dose is not automatically reported by the Ethos software to ARIA. Therefore, “manual simulation” of each treatment session is required in ARIA for dose reporting purposes and, thus, correct documentation. This step was not reliably performed in at least 27 cases over one year. We implemented checks as part of the morning check QA to identify these lapses. We observed instances where a missed fraction was mistakenly simulated in ARIA on the subsequent treatment day, intended as a correction. This error is especially difficult to detect.

The transfer of patients to another treatment machine proved particularly challenging in terms of documentation and demanded significant resources. We also noticed that due to two different software versions, the Ethos allowed for a smaller cranio-caudal delta couch shift from the localization point to the isocenter of a Halcyon machine with software of a lower version.

In some cases, plans could not be loaded on the treatment machine, although QA was possible. This issue was identified shortly before the first fraction. These plans, often for breast treatments, required a re-import after excluding organ at risk structures that led to the described software issues. None of these plans could be imported into Mobius either, which would have been helpful in identifying such plans. Resolution of this issue is anticipated in the next software release.

## Discussion

During the introduction of a new treatment technique in an institution, it will always be possible to observe a learning curve, which will be present despite training and thorough preparation. Therefore, a previously performed prospective risk analysis for the adaptive workflow was updated by incorporating clinical experience.

The radiotherapy treatment workflow changes dynamically, especially for a newly introduced technique such as adaptive radiotherapy. This study supports that re-evaluating a risk analysis enables adaptation to workflow changes and inclusion of additional measures. For a completely new treatment machine and modality in our department, substantial additions were made after the first year.

The primary risk (TOP 1) emerged when the physician usually assigned to adaptive treatments with in-depth knowledge of a patient was unexpectedly unavailable, and another physician with training on the machine but less specific knowledge of the contouring details of the individual patient had to take over. Comprehensive and structured documentation of contour details can greatly reduce the associated risk. Ideally, adaptive sessions should be performed by consistent team members, e.g. radiation oncologists with in-depth knowledge of the patient. At the same time, there should be a strategy for (spontaneous) needs for replacement of personnel acquainted with the individual. Involvement of the radiation therapists, who are generally available at the machine for prolonged time periods, is a possibility.

Insufficient automatic contouring leads to the need for individual contouring and, therefore, increases the likelihood of errors in manually adjusted contours (TOP 15). We observed automatic contouring to struggle adapting planning target volumes (PTV) directly, requiring lots of manual edits. Consequently, we favour the contouring of individual clinical target volumes (CTV) and lymph nodes and then using the software function to mathematically derive the PTV anew during each adaptive session using the respective margins.

Many of the highest ranked risks are associated with treatment planning using templates (TOP 5, 7, 9, 12, 13 and 19). Adaptive radiotherapy demands robust planning parameters capable of yielding good plans not only on the planning CT dataset, but also on the anatomy varying daily. Templates should be thoughtfully prepared well in advance for new treatment sites and regularly updated. Dose constraints for all relevant organs at risk should be clearly communicated and meticulously documented before planning. Providing universal access to an emulator would enable users to test and evaluate templates *in silico*.

While the workflow can benefit from further refinement based on this analysis, some aspects require intervention by the vendor. With better imaging solutions and Ethos 2.0 currently being rolled out, many of the interconnectivity issues between the Ethos software and ARIA (TOP 2 and TOP 6), as well as misassigned densities in the synthetic CT (TOP 14, TOP 16) are being addressed. Pitfalls in the software use (TOP 10 and TOP11) should be addressed and resolved. With the current version, longer machine or software faults and the subsequent irradiation of the patients on a different treatment machine, require a lot of manual simulation to ensure accurate fraction counting in both Ethos and ARIA to avoid irradiation of a wrong number of fractions, which is anticipated to be resolved with future software updates. The use of the synthetic CT is a major risk, particularly when anatomical changes occur in regions with large density differences, as documented in the literature [18], [19]. Utilizing CBCT for dose calculation or secondary dose calculation could mitigate gross errors. Yet to be addressed are the usability and accessibility of electronic portal imaging, especially for in vivo dosimetry, and the seamless integration of third-party quality assurance for vendor-independent quality assurance.

In the initial analysis, the highest ranked failure mode (Rank 1) was an incomplete verification of essential plan parameters by the physicist upon plan acceptance. In this context, we have previously outlined the challenges in compiling a comprehensive list of check parameters for a new workflow. The recorded irregularities include several unexpected failure modes (for example derived structures turned into static contours, target volume exceeding the capacity of a single isocenter), and these could be incorporated to the checklist. Additionally, a wrong plan was inadvertently sent to Mobius for QA during the standard IGRT treatment (Rank 9) and phases were not correctly entered into the Ethos software, resulting in plans not being timely or correctly sequenced (Rank 15). Additionally, literature data on specific aspects became available in the meantime, such as a comparison of secondary dose calculation and measurement-based quality assurance. [20]

In many countries, radiation therapists play a significant role in the delivery of adaptive treatments. The re-evaluation suggests that a well-structured procedure, involving a limited number of experts who have comprehensive knowledge of the patient, is desirable, particularly for timely and concise treatments aimed at reducing intrafractional anatomical changes. These aspects become increasingly important when margins are narrowed in adaptive treatments. Based on department-specific experience, radiation therapists-only workflows are expected to streamline the workflow. We acknowledge that it may be different in institutions where radiation therapists interchange between treatment machines more often or work in shifts.

Due to the nature of the Ethos software, workflows on this machine will be mostly comparable across different institutions. Especially the more technical aspects in the workflow should be universal. However, the use of specific equipment, such as intrafractional tracking devices and additional software, workflows or the distribution of tasks certainly varies between institutions, such that an individual risk analysis for the specific setting is necessary. A number of mitigation strategies are presented, but this list is by no means complete.

Many of the irregularities reported within the first year of using the Ethos machine were predominantly related to the IGRT workflow. With the integration of data from Eclipse into the Ethos software, the workflow is prone to errors at this step. While many irregularities only occur due to the import step used in this institution, the choice of a different workflow surely introduces different risks. Therefore, performing a systematic risk analysis for the non-adaptive workflow should be considered by new users of the system as well.

The risk analysis profited from the structured evaluation using the format of the dedicated software. It was particularly helpful to systematically examine both existing and added preventions and barriers. A key outcome of the repeated risk analysis includes updates to the standard operating procedures, detailing necessary checks at various stages to ensure that observed irregularities are not repeated. Implementing automatic checks within the Ethos software, similar to those in Eclipse with scripting or third-party software, would be advantageous.

## Conclusion

The prospective risk analysis prior to commissioning of online-adaptive treatments on a new treatment machine proves critical in developing a safe workflow and has its justification. However, a prospective analysis without in-depth experience with a treatment technique or a machine cannot predict all possible scenarios. All irregularities within the first year of adaptive treatments, restricted to the pelvic region, were recorded and served as the foundation for a re-evaluation. Awareness of these issues and minor modifications to the initially designed workflow further mitigated risks in adaptive treatments. However, with upcoming technological changes and adaptation programmes targeting a larger variety of anatomical regions, it can be expected that known risks will be perceived differently and further risks will be identified.

## CRediT authorship contribution statement

**Sonja Wegener:** Conceptualization, Investigation, Project administration, Writing – original draft. **Paul Käthner:** Investigation, Writing – review & editing. **Stefan Weick:** Investigation. **Robert Schindhelm:** Investigation, Writing – review & editing. **Kathrin Breuer:** Investigation. **Silke Stark:** Investigation. **Heike Hutzel:** Conceptualization, Investigation. **Paul Lutyj:** Writing – review & editing, Investigation. **Marcus Zimmermann:** Investigation, Writing – review & editing. **Jörg Tamihardja:** Investigation, Writing – review & editing. **Andrea Wittig:** Resources, Writing – review & editing. **Florian Exner:** Conceptualization, Project administration, Writing – review & editing. **Gary Razinskas:** Conceptualization, Investigation, Writing – review & editing.

## Data Availability Statement

Data can be made available on request. No patient data was used for this study.

## Declaration of competing interest

The authors declare that they have no known competing financial interest or personal relationships that could have appeard to influence the work reported in this paper.
