# Supplementary material for: Re-evaluation of the prospective risk analysis for artificial-intelligence driven cone beam computed tomography-based online adaptive radiotherapy after one year of clinical experience
Source: Z Med Phys. 2024 Jun 8;34(3):397–407. doi: 10.1016/j.zemedi.2024.05.001 (PMC11384936; doi:10.1016/j.zemedi.2024.05.001)
Supplement: Supplementary Data 1 [file mmc1.docx]

**Appendix A**

Table 1 Scoring guidelines for assessing severity, occurrence, and detectability.

| **Score** | **Severity S** | **Occurrence O** | **Detectability D** |
| --- | --- | --- | --- |
| **1** | no or negligible effects | once every 5-30 years | stops process or is always detected within process step |
| **2** | temporary impairment | once every 1-5 years | detected in subsequent step |
| **3** |  |  |  |
| **4** | impairment requires prolonged treatment with unclear consequences | monthly to yearly | systematic technical inspection along with manual check |
| **5** |  |  | four-eyes inspection as per specific checklist |
| **6** |  |  | four-eyes inspection, unspecified |
| **7** | permanent impairment of body function, incident report required^+^ | weekly to monthly | two-eyes inspection |
| **8** |  |  | not systematically checked |
| **9** | death within a short time frame | daily to weekly | may be detected by chance |
| **10** |  |  | unlikely to be detected |

*^+^ This includes all incidents mandated for reporting according as per German law § 108 StrlSchV, as stated in its Appendix 14 and summarized in reference [17]. Pertinent for this table are variations exceeding 10% or 4 Gy in the planned dose for organs at risk or target volumes, treatment durations prolonged by more than a week, irradiation of an incorrect patient, or use of an incorrect treatment plan.*
